# Natural Gums to Improve the Physicochemical Stability of Cake Creams

**DOI:** 10.3390/foods10102261

**Published:** 2021-09-24

**Authors:** Oscar López-Balladares, Patricio J. Espinoza-Montero, Ramiro Acosta-Sandoval

**Affiliations:** 1 Escuela de Ciencias Químicas, Pontificia Universidad Católica del Ecuador, Quito 170525, Ecuador; ohlopez@uce.edu.ec (O.L.-B.); reacosta@uce.edu.ec (R.A.-S.); 2 Facultad de Ciencias Química, Universidad Central del Ecuador, Quito 170521, Ecuador

**Keywords:** cake cream, natural gums, rheology, stability, syneresis

## Abstract

The physicochemical properties of pastry and confectionery products greatly influence the aesthetic design of a cake topping, since they can be susceptible to physicochemical changes in a very short time, so maintaining a good appearance and texture of the topping becomes a challenge. Generally, cake creams deteriorate over time. The evaluation of the physicochemical properties of natural gums (arabic gum, tara gum, carrageenan, and pectin) is proposed in this work as a way to improve the physicochemical stability of butter-based cake creams (coverage creams) to maintain the initial appearance of the cream and to lengthen the separation time of their phases. For this purpose, some parameters related to the physicochemical stability of the cream, such as viscosity, density, bubble size, syneresis, volume and rheological behavior were measured. The result of the ANOVA and Tukey’s tests displayed significant differences for the measured parameters, which shows that natural gums substantially improve the stability of butter cream. The best natural gum found was the tara gum (TG) which improved viscosity 5.6 times with respect to that of the cream without gums ($\bar{\eta }$ without gums = 15.49 Pa·s, $\bar{\eta }$ with TG = 87.09 Pa·s), while the bubble size remained small, 1.6 times smaller compared to that of the cream without gum ($\bar{BS}$) without gums = 57 μm, ($\bar{BS}$) with TG = 35 μm), and the volume loss decreased two times when compared to that of the cream without gums (($\bar{\Delta V}$) without gums = 1.57 cm^3^, ($\bar{\Delta V}$) with TG = 0.80 cm^3^). The cream with TG showed better rheology compared to that of the cream without gums (the cream without gums exhibited a plastic and thixotropic behavior, with permanent elastic deformation, while cream with TG exhibited thixotropic behavior without permanent elastic deformation). Finally, it was found that the cream with TG acquired a higher thixotropic index (TI) compared to that of the cream without gums (TI max. without gums = 17.40 y 71.78 q.u., TI max. with TG = 74.67 and 1559.90 q.u., at 4 °C and 25 °C, respectively) which demonstrates the effective contribution of cream with TG in 66.67% of the measured parameters.

## 1. Introduction

Coverage creams in the pastry and confectionery industry play a very important role in giving softness, texture and presentation to cakes, pies, and desserts. In Ecuador, the pastry sector combined with the bakery sector, for which there are around 5679 businesses, generates sales of USD 306 million per year [1]. 

There are cases of creams that cease to have value due to their physicochemical instability—in most cases yielding a phase separation—without being affected by considerable microbiological contamination. The result is a flaccid body of less volume and mass, with alterations in its texture and viscosity (η) characteristics, which makes the cake unattractive to the consumer [2].

Several studies have been carried out in relation to the evaluation of stabilizers in colloidal systems made up of karaya gum, carob gum and guar gum for ice cream, showing that ice creams formulated with 50% carob gum and 50% guar gum have a lower percentage of melting and less time to the fall of the first drop compared to the control sample. Additionally, stability studies that compared concentrations of 0.3, 0.35 and 0.4% (*w*/*w*) of a 50% carob gum and 50% guar gum mixture against a control sample that had a commercial stabilizer applied at 0.3% (*w*/*w*) have shown that the ice cream with 0.4% (*w*/*w*) showed greater rubberiness, while the control sample showed a greater roughness [3]. On the other hand, comparative studies between gellan gum and pectin to form fluid gels in fermented milks were carried out to test the viscosimetric and stabilizing properties when compared with that of a sample that did not contain hydrocolloids, in which no fluid gel was formed. The experimental findings proved that, unlike pectin, gellan gum was capable of creating significant yield strength values which improved the stability of colloidal particles and extrindry solid particles added in the milk drink. However, pectin improved the stability when it was combined with gellan gum. The formation of the fluid gel can be attributed to the permanent interactions that occur between gellan gum and milk proteins, forming gels of “hairy particles” as transient interactions between gelled particles [4].

In the case of creams, it is usual to extend their shelf life by adding modified starch from corn, rice, potato or wheat [5,6]. However, the limitations of use are the properties of application in terms of pH, temperature and amount of water, since they frequently do not provide the desired results in the final product due to variations in the process conditions and the raw materials used for each formulation [5].

To prevent the coalescence and flocculation of the colloidal particles found in the food matrix, certain compounds (known as stabilizants) can be added to certain foods. In this context, gums (which are polysaccharides of a very high molecular weight) have the ability to increase the viscosity of the colloidal system, and can also act as cryoprotectants [6]. Additionally, they have the ability to retain, swell and gel in the presence of heat. Due to the tendency of gels to partially separate in the liquid phase accompanied by a decrease in the volume of the system, it is important to evaluate the volume and syneresis [7].

It is also pertinent to assess parameters such as the density and bubble size of the cake cream, since it is a food product obtained by whipping processes, for which its matrix has some air incorporated and therefore it is necessary to measure the foam stability; this depends on factors such as Ostwald maturation, which is the diffusion of gas from the smallest bubbles to the largest ones, the coalescence of the bubbles due to the instability of the film that separates them, and the drainage of liquid of the foam layer due to gravity, which in turn is summarized in the excessive thinning of the lamellae inducing the rupture of the bubbles [8,9].

Another parameter for measuring the stability of colloidal systems is the rheological behavior, where thixotropy is a relevant property in terms of the firmness and softness of the creams. It occurs when the changes in the microstructure are reversible, which are time-dependent and expressed by the effect of shear stress (τ). This effect is an ideal situation that occurs under certain experimental situations, in comparison with the numerous situations in which food, in its state of flux, presents irreversibility due to its partial or total decomposition of the structure of the food matrix [10]. Likewise, the thixotropic index (TI), a parameter that allows for evaluating the degree of rupture of the structure, has allowed in previous investigations, such as the one studied by Lavaselly and Rasia (2004) [11] in emulsions, to identify that there is a correlation between thixotropic properties and viscosity, where it is stated that TI maintains a relationship with the physical stability of colloidal systems [11]. 

Uysal et al. [12] carried out a study related to the rheological behavior of colloidal food systems, in which pastry creams containing pasteurized liquid whole egg (LWE) were evaluated versus control group creams containing unpasteurized LWE. Specific density, foamability and rheological measurements (shear rate vs. shear stress and shear rate vs. viscosity) of the cake creams were performed. As a result, a pseudoplastic behavior was observed in all cream formulations indicating a decrease in apparent viscosity as a function of increasing shear rate [12]. An increase from 0.29 to 0.49 in cream specific densities was observed as LWE pasteurization parameters [12]. Furthermore, a decrease in the foamability of creams with LWE was observed as the pasteurization parameters of LWE increased [12]. The evaluation of the rheological characteristics of the cream for cakes is important because it provides a previous knowledge about the structure of the baked cakes that, together with the density and foamability, demonstrate the importance of its measurement in the stability of colloidal food systems.

The objective of this work was to evaluate the physicochemical properties of coverage creams for cake, considered as a lyophobic colloidal system (little attraction between the dispersed phase and the dispersant phase), in which polysaccharide-type hydrocolloids such as natural gums were used to reduce the separation of phases of the cream. Various gums from different plant sources, such as Pectin Gum (PG), Tara Gum (TG), Arabic Gum (AG), and Carrageenan Gum (CG) were studied to establish which of these works best as a stabilizer for cake cream, considering the temperature conditions in which the product is going to be stored, i.e., at room temperature and in refrigeration.

## 2. Materials and Methods

The materials and equipment used were: Bohlin Instruments CVO rheometer, analytical balance of appreciation 0.0001 g, optical microscope (Nikon Eclipse E100, Nikon Instruments Inc., Melville, NY, USA), refrigerator, drying oven (Memmert brand, Memmert GmbH + Co. KG, Germany), centrifuge (Danon/IEC HN-SII Centrifuge, American Laboratory Trading, INC., San Diego, CA, USA), mixer (Umco brand, 7 speeds, UMCO S.A., Ecuador), thermometer, stopwatch, hand refractometer (BRIX/ATC brand, Northern Brewer, LLC., United Kingdom), neubauer chamber, Vernier caliper of appreciation 0.01 cm, falcon tubes of 50 mL, graduated cylinders of 100 mL, and syringes of 10 mL.

### 2.1. Additive Substances

The used additives were natural gums: pectin (unipectine RS 150 ci-trus, Cargill Texturizing Solutions, Saint-Germain-en-Laye, France) acquired from the Casa de los Químicos in Quito; tara gum (tara gum HV, Silvateam, Lima, Peru), obtained from CO-DAN in Quito; gum arabic (gum arabicum medium light, Alpha Trading GMBH, Hamburg, Germany), acquired at the Casa de los Químicos, Quito; carrageenan (carra-geenan MCH 5316, Gelymar, Santiago, Chile) obtained at ADITMAQ in Quito.

### 2.2. Elaboration of Coverage Creams 

The coverage creams for cake were made as reported by De la Traba Luis and García Víctor [13]: first, preparation of a syrup with sugar and water, between 67° and 69° Brix; second, beating the yolks for 2 min, after which the syrup was added and the mixture was stirred for 2 additional minutes.

The gums were incorporated by two methods. For method 1 (dissolved gum), gum solutions were made in water at 70 °C (1:9) by stirring for 30 s, followed by adding the gum solution to the yolk–syrup mixture and then mixing for 2 min, finally adding the butter and whipping for another 5 min. A concentration of 0.8% by weight was used for all creams, thus preventing the amount of additive from exceeding 1% of the permissible limit of most gums [14]. For method 2 (powdered gum), first the syrup was prepared with sugar and water, between 67 and 69° Brix; second, the yolks were beaten for 2 min, after which the syrup was added and the mixture was stirred for a further 2 min. Then the gum powder was added to the mixture of yolks and syrup and stirred for 2 min while adding the butter, finally beating the mixture for a further 5 min.

### 2.3. Evaluation of Visual Acceptability of Cake Creams

The samples were coded for sensory evaluation by 20 untrained judges, which consisted of a preference test between four pairs of creams made with the four gums by the two methods proposed [15]. Each judge was presented with a pair of creams made with the same gum by the two methods, and they were asked to choose one of the two creams according to consistency. The process was repeated for the other three pairs of creams made with the remaining gums. For each pair, an answer was obtained for a total of 80 results.

### 2.4. Characterization of Accepted Cake Cream by Physicochemical Parameters

The characterization of the cake cream that was accepted by the judges was carried out in triplicate at room temperature (at 25 °C) and refrigeration (at 4 °C), in different storage periods of up to 28 days: four with the gums added to a concentration of 0.8% and one without presence of gums (blank). See Table 1. A total of 270 tests were carried out for the parameters: viscosity, density, bubble size, volume and syneresis.

#### 2.4.1. Viscosity 

Viscosity was measured using the “Bohlin Instruments CVO” rheometer, with measuring system PP20 (20 mm parallel rotating plate), GAP of 1000 (distance between the turntable and the fixed plate of 1000 µm, that is, the height of the sample during the measurement) at a constant velocity gradient (D) 0.86 s^−1^, time of 7.2 s and temperature of 25 °C [16]. Three repetitions were carried out for each formulation of the creams, at the two temperatures and for each storage time (0, 3, 7, 10, 14, 17, 21, 24, 28 days); the average is reported in all cases.

#### 2.4.2. Density

The density (ρ) was computed from the mass and the volume of the sample in containers of approximately 16 cm^3^. Three repetitions were carried out for each formulation of the creams, at the two temperatures and for each storage time (0, 3, 7, 10, 14, 17, 21, 24, 28 days); the average is reported in all cases.

#### 2.4.3. Bubble Size

The bubble size (BS) was measured in a Nikon Eclipse E100 optical microscope using the neubauer camera with a 10× and a 40× observation lens, depending on the size of the bubble. A corresponding photograph was taken to later measure the length of the bubble using an appropriate scale. The photographs were taken and processed using the Leica LAS X software (Leica Microsystems, Germany), which was connected to the Nikon Eclipse E100 optical microscope (Nikon Instruments Inc., Melville, NY, USA). The bubble diameter was measured in triplicate of three expanded samples on the neubauer chamber plate for each formulation, temperature, and storage time (0, 3, 7, 10, 14, 17, 21, 24, 28 days). A total of 270 images were obtained.

#### 2.4.4. Volume Variation

The volume change (ΔV) of the creams was measured with a vernier caliper with an appreciation of 0.01 cm. The samples were placed in 100 mL graduated cylinders up to a height of 17 cm with the subsequent measurement of the distance between the initial height and the height of each of the samples at various storage times. The internal diameter of the graduated cylinder was also measured using the same caliper. The ΔV was obtained by Equation (1). Three repetitions were carried out for each formulation of the creams, at the two temperatures and for each storage time (0, 3, 7, 10, 14, 17, 21, 24, 28 days); the average is reported in all cases.

ΔV = πR^2^ΔH (1)

where ΔH = height variation, R = internal radius of the graduated cylinder. 

#### 2.4.5. Syneresis

Syneresis (S) was measured by the modified technique of Ginee et al. (Guinee et al., 1995) [17], which consists of weighing the sample in a falcon tube and centrifuging it at 3000 rpm for 30 min (for this a Danon/IEC HN-SII centrifuge was used); once the phases were separated, the liquid phase was weighted and the percentage of syneresis (% S) was calculated by means of Equation (2) [18]. Three repetitions were carried out for each formulation of the creams, at the two temperatures and for each storage time (0, 3, 7, 10, 14, 17, 21, 24, 28 days); the average is reported in all cases.

(2) $$\%\;S=\frac{\mathrm{mass}\;\mathrm{of}\;\mathrm{liquid}}{\mathrm{sample}\;\mathrm{mass}}\times 100$$

#### 2.4.6. Rheology

The rheology was evaluated by measuring the shear stress and the viscosity of the five creams, using curves of τ vs. D and η vs. D, where τ is expressed in Pascal units (Pa) and D is expressed in (cm/s)/cm or s^−1^, in a “going” and “return” process with the “Bohlin Instrument CVO” rheometer, using a PP 20 measuring system, GAP of 1000, at 25 °C temperature and a variable speed gradient in the range of 0 to 15 s^−1^, for a total time of 240 s [16]. In addition, the area between the “going” and “return” curves was measured, for which the Bohlin software was used; this area corresponds to the hysteresis cycle and is called the thixotropic index which is expressed in quadratic units (q.u.). The rheological study was run in large storage time intervals: 0, 7, 14, 21 and 28 days. Three repetitions were carried out for each formulation of the creams, at the two temperatures and for each storage time. A total of 150 tests were carried out for the rheological parameters, the average is reported in all cases.

## 3. Results

### 3.1. Results of the Acceptability Evaluation of the Creams 

The results of the visual acceptability of the cake creams made with natural gums by addition in solution (M1) and powder (M2) are shown in Table 2.

The results obtained from the 20 judges in the sensory evaluation of the four pairs of creams made with the four natural gums (TG, AG, CC, PG), both by M1 and M2 (total 80 trials performed), showed a trend towards the preference of creams made by method 2, which indicates a better appearance in terms of texture and creaminess of the creams, that is to say, creams that presented greater homogeneity of the ingredients. According to Roessler et al., statistically, for 80 trials, the concordant minimum for a two-tailed test is 52, which indicates that 69 judgments in favor of method 2 is a significant result with a confidence level of 99% [19]. Therefore, there is a significant difference in the appearance of the creams. As a consequence of this result, all subsequent creams were prepared by method 2 for the evaluation of physicochemical parameters.

### 3.2. Results of Characterization of Accepted Cake Cream by Physicochemical Parameters

**Figure 1:**
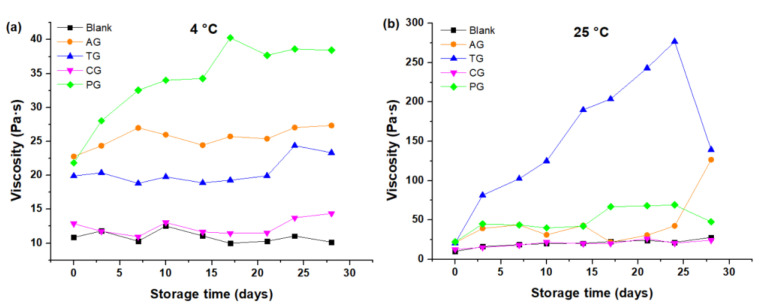
Viscosity as a function of storage time: (**a**) Creams stored at 4 °C; (**b**) Creams stored at 25 °C.

The different creams showed that η increases with storage time both at 4 °C and at 25 °C; cream made with PG stored at 4 °C had a greater effect in relation to other creams (see Figure 1a). Although the cream with PG also behaved similarly at 25 °C, a drastic effect appeared in the cream with TG, which was the cream with the highest η of all (Figure 1b). The results shown in Figure 1 indicate that the η of the creams was greater at 25 °C in relation to that of the creams stored at 4 °C, regardless of the gum used.

**Figure 2:**
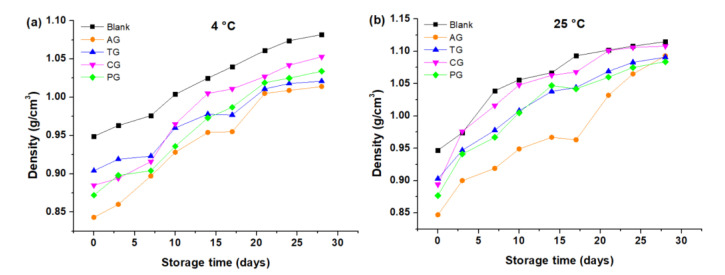
Density as a function of storage time: (**a**) Creams stored at 4 °C; (**b**) Creams stored at 25 °C.

The ρ increased with storage time both at 4 °C and at 25 °C. In this sense, creams made with AG stored at 4 °C and 25 °C kept a lower ρ than the rest of the creams at all times; see Figure 2a,b. At 25 °C, the creams made with PG and TG had very similar densities at each time (see Figure 2b). The results shown in Figure 2 indicate that the ρ of the creams was greater at 25 °C in relation to that of the creams stored at 4 °C regardless of the gum used.

**Figure 3:**
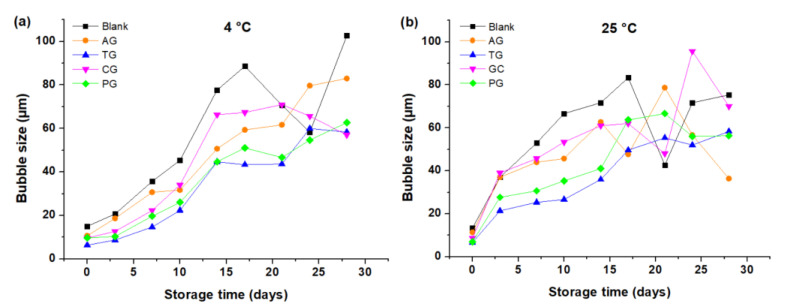
Bubble size depending on the storage time: (**a**) Creams stored at 4 °C; (**b**) Creams stored at 25 °C.

**Figure 4:**
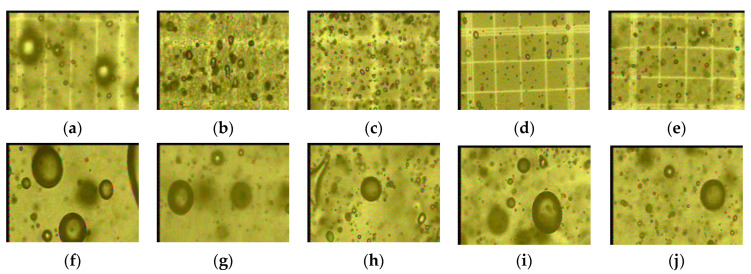
Creams observed with a 40× objective lens on day 0: (**a**) Blank cream; (**b**) cream with AG; (**c**) cream with TG; (**d**) cream with CG; (**e**) cream with PG. Creams observed with a 40× objective lens on day 21 at 4 °C: (**f**) Blank cream; (**g**) cream with AG; (**h**) cream with TG; (**i**) GC cream; (**j**) cream with PG.

The BS increased for all creams, until approximately 14 and 17 days of storage at 25 °C and 4 °C, respectively (see Figure 3a,b). The creams made with PG and TG maintained a similar behavior throughout the storage time at 4 °C, and their bubble diameters were smaller than the rest; see Figure 3a. At 25 °C, the creams made with TG maintained a lower BS than the rest of the creams for the entire storage time; see Figure 3b. Figure 3 indicates that the BS of the creams with AG, PG and TG was higher at 25 °C. Some photographs at time zero show that the BS was higher in creams that did not have additives with respect to those that incorporated gums (see Figure 4a–e). As the storage time increased, the bubbles increased in size (see Figure 4f–j) until they broke, generating a lower amount of air—especially in creams without gums when compared with those that had a stabilizer—as evidenced on day 21 at 25 °C.

**Figure 5:**
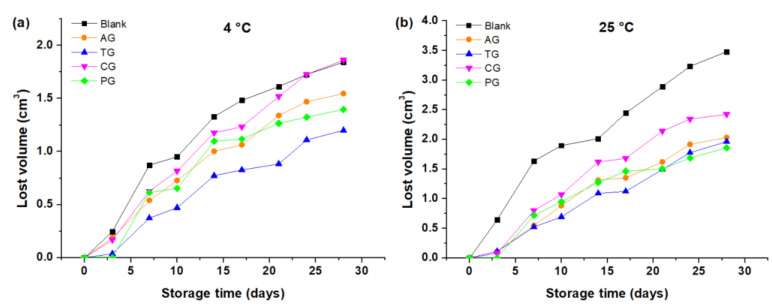
Lost volume as a function of storage time: (**a**) Creams stored at 4 °C; (**b**) Creams stored at 25 °C.

**Figure 6:**
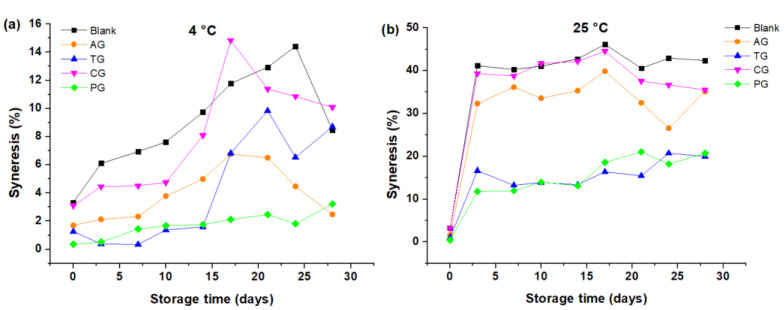
Syneresis as a function of storage time: (**a**) Creams stored at 4 °C; (**b**) Creams stored at 25 °C.

The ΔV increased with storage time at both 4 °C and 25 °C; in other words, the cream lost more volume as the storage time increased. As shown in Figure 5a,b, the creams made with CG and without gums lost the most volume, while creams made with TG were those that lost the least volume for the entire storage time at 4 °C (Figure 6a). At 25 °C, the creams made with PG, TG, and AG exhibited a similar behavior for the entire storage time (Figure 5b). Figure 5 indicates that the ΔV of all the creams was greater than 25 °C in relation to that of the creams stored at 4 °C on average over 28 days of storage.

The % S of the creams made with AG, PG, CG, and without gums tends to increase until approximately day 17 and then the value decreased at both 4 °C and 25 °C (Figure 6a,b). Additionally, at 4 °C the cream made with PC exhibited less syneresis in relation to that of the rest of the creams for 28 days (Figure 6a), while at 25 °C the creams made with PG and TG showed less syneresis in relation to that of the rest of the creams, in which large differences in syneresis values were observed (Figure 6b). The results shown in Figure 6 indicate that the % S of all the creams was higher at 25 °C in relation to that of the creams stored at 4 °C as an average of the 28 days of storage.

The Analysis of Variance (ANOVA) was performed with the statistical program “IBM SPSS Statistics 25”, using a significance level of 0.05 for the completely randomized design with factorial arrangements A × B × C: formulation (A) temperature (B) and storage time (C). From this significant difference the Tukey test was performed through multiple comparisons for the formulation factor (A) which was the most interesting in the research. The estimated marginal means evaluated in the Tukey test are described in Table 3 for viscosity ($\bar{\eta }$), for density ($\bar{\rho }$), for bubble size (($\bar{BS}$)), for lost volume (($\bar{\Delta V}$)) and for syneresis ($\bar{S}$).

The ANOVA showed a significant difference for the measures of η, ρ, BS, ΔV and% S for the 28 days exhibited.

The results of the Tukey test showed different concordances between the formulations. The η of the cream made with CG was similar to that of the white cream (subgroup 1), and there was no significant difference between these two; the η of the cream made with GA was similar to that of the cream made with PG (subgroup 2), and the η of the cream made with TG (subgroup 3) was different from that of the creams of subgroup 1 and 2, that is, there was a significant difference between all samples. For density they indicated that the ρ of each of the creams made with AG, PG, TG, CG and without gums were different; that is, there was a significant difference between all samples. The BS of the cream made with TG was similar to that of the cream made with PG while the BS of the cream made with AG was similar to that of the cream made with PG, but different from the cream made with TG; additionally, the BS of the cream with CG was similar to that of the cream made with AG, but different from that of the creams made with TG and PG, and the BS of the white cream was similar to that of the cream made with CG but different from that of the creams made with TG, PG and AG. The ΔV of the cream made with PG was similar to that of the ΔV of the cream made with GA (PG and GA subgroup 2), so there is no significant difference, while the ΔV for the creams made with TG (subgroup 1), CG (subgroup 3) and white cream (subgroup 4) were different from each other and also from those of subgroup 2. Regarding syneresis, the% S values of the creams made with PG, TG, AG, CG and without gums were different from each other due to the presence of significant difference between all samples.

#### Rheology Results 

The rheological determination was based on the principle of non-Newtonian fluids, in which by applying shear forces to the different samples, they modified their viscosity, becoming more fluid. In this sense, if the change in viscosity does not depend on the elapsed time, we have the steady state systems, within which we can mention the pseudoplastics, plastics and dilatants whose fluidity curves are reversible. Pseudoplastics decrease their viscosity and dilatants increase their viscosity as D increases, while plastic systems decrease viscosity, but, unlike pseudoplastics, they require minimal shear stress to begin to flow. If the viscosity of the system, in addition to changing with the value of D, also changes with time, we have time-dependent systems, which can be thixotropic or rheopexic, whose fluidity curves are irreversible since the “going” curves and “return” curves do not coincide, forming a hysteresis between them (the area between the going and return curves). Thixotropics decrease their viscosity and rheopexics increase their viscosity as D increases, along with the formation of the hysteresis mentioned. 

**Figure 7:**
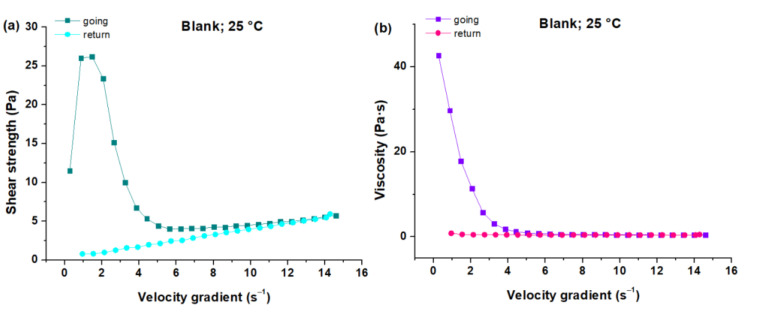

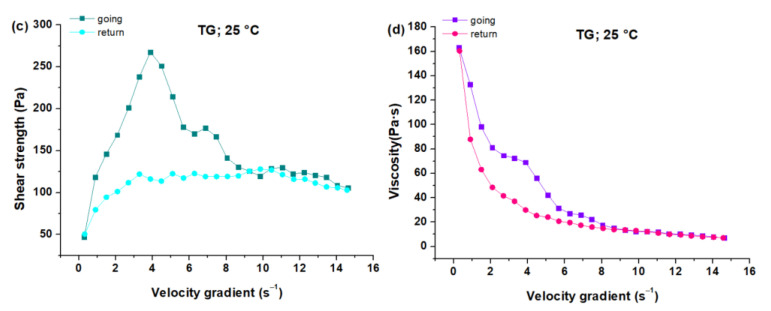
Shear stress curves as a function of the velocity gradient of the creams stored at 25 °C on day 28: (**a**) τ vs. D of the blank cream, (**c**) τ vs. D of the cream with TG. Viscosity curves as a function of the velocity gradient of the creams stored at 25 °C on day 28: (**b**) η vs. D of the blank cream, (**d**) η vs. D of the cream with TG.

The results of the rheograms showed that in creams at 4 °C on day 28, as D increased, a greater τ was needed. The creams with TG, AG and PG had higher hysteresis than that of blank creams and creams with CG, and as D increased, η decreased. The observed “return” curve maintained linearity up to a speed gradient of approximately 4 s^−1^ for blank cream and cream with CG, in contrast with creams containing AG, TG and PG, which maintained linearity up to approximately 6 s^−1^. In this sense, on day 28 at 4 °C, blank creams and creams with CG had a tendency towards plastic behavior, while TG, AG and PG creams had thixotropy. At 25 °C and on day 28, there were variants with respect to the cooling process: in the “going” curve, as D increased, τ increased at the beginning, then decreased, and finally increased again. This is observed in all creams except for the cream with TG, where τ increased at the beginning and then decreased. In addition, on day 28 at 25 °C, as D increased, η decreased, from which hysteresis is observed with the generation of permanent elastic deformation in creams with CG, AG, PG and blank cream (see Figure 7a,b), unlike the cream with TG which showed recovery after deformation (see Figure 7c,d). In this sense, on day 21 at 25 °C, all creams had a tendency towards thixotropic behavior.

Table 4 shows the results of the rheological behavior of the creams, being plastic or thixotropic, which depends on the storage temperature, the storage time and the type of gum used for the preparation of the cream. In the case of blank cream and the cream made with CG, at 4 °C their behavior was plastic, while at 25 °C it experienced a thixotropic behavior. In addition, creams made with AG, TG and PG had a thixotropic behavior at the two storage temperatures; unlike the other two creams. The cream sample that contained AG showed a thixotropic behavior from day zero, while from day 21, creams stored at 25 °C—except for the one containing TG—showed a permanent elastic alteration after being subjected to a rheological process; see Table 4.

**Figure 8:**
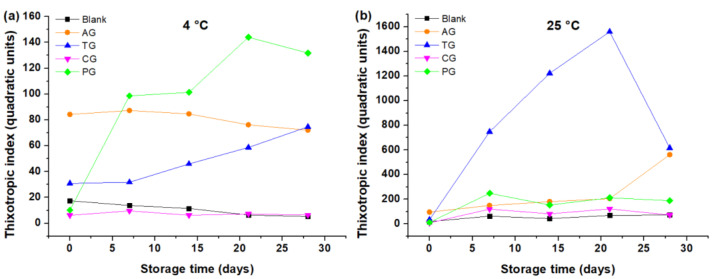
Thixotropic index as a function of the storage time of cake creams. (**a**) Creams stored at 4 °C; (**b**) creams stored at 25 °C.

The thixotropic index of the different creams at 4 °C showed a greater hysteresis for the samples that contained PG and AG (see Figure 8a). In the case of blank cream, the TI decreased from 17.40 q.u. down to 5.25 q.u., while for creams with TG and PG it increased from 30.89 to 74.67 q.u. and from 10.17 q.u. at 131.82 q.u., respectively. Meanwhile, the cream with AG tended to lower its TI; however, the values are relatively large, from 84.25 to 72.05 q.u. In addition, for the cream with CG the values remained low around 6 q.u. for the 28 days of storage. On the other hand, at 25 °C the TI of the blank cream increased from 18.33 to 71.78 q.u., while the creams with GC and PG had a local maximum TI of 121.10 q.u. at 21 days and 247.74 q.u. at 7 days, respectively. The results also showed that the cream with TG tended to increase its hysteresis in a relevant way as a function of time until day 21 (from 33.40 to 1559 q.u.), and from this day the TI decreased to 616.59 q.u. There was a difference presented by the cream with GA, since its hysteresis increased sharply from day 21 until it reached a value close to that of the cream with TG in the 28 days of storage (from 204.08 to 561.23 q.u.); see Figure 8b. Finally, a greater hysteresis can be observed in samples stored at 25 °C than in those stored at 4 °C; see Figure 8a,b.

In Table 5, a summary of the parameters measured considering the needs of the stabilizing properties and the significant differences in the obtained results is shown. This is indicative that the AG, TG and PG gums aid to stabilize the cream, while the CG does not contribute significantly to reducing the phase separation of the cake cream. The results of Table 5 are equivalent to the percentage of compliance with the total of the measured parameters: AG 16.67%, TG 66.67%, PG 33.33% and CG 0%

## 4. Discussion

As can be seen in Table 3, in which the average values of the evaluation of physicochemical properties up to 28 days, both at room temperature and refrigeration, can be seen, tara gum provides a higher viscosity ($\bar{\eta }$ of TG = 87.09 Pa·s) than the rest of gums and is obviously higher than that of the white cream ($\bar{\eta }$ of blank = 15.49 Pa·s), which is interpreted as a gum capable of thickening and stabilizing the ingredients of the covering cream. The increase in viscosity can be explained by the fact that gums need time to interact with the phases of the cream and to be able to form a three-dimensional network of macromolecules [20]. Furthermore, the magnification is different for each gum due to the differences between their chemical structure [9,20,21]. Moreover, the marked increase in the viscosity of the cream containing TG at 25 °C indicates the formation of a gel as the storage time elapses.

The addition of the gums influenced the density of the creams, causing a spongier texture, due to the inhibition of the loss of incorporated air. The increase in density can be explained by the fact that butter creams lose volume due to separation of the phases of the system. Furthermore, as the temperature increases, this decrease in volume is more pronounced. The cream made with AG had the lowest density, probably due to that during its elaboration it is capable of incorporating and retaining a large amount of air, which causes the same volume to have less mass and therefore density [22]. In Table 3, the average value of densities is verified during the 28 days of storage for the two temperatures ($\bar{\rho }$ of AG = 0.9555 g/cm^3^) and secondly for the pectin cream ($\bar{\rho }$ of PG = 0.99 g/cm^3^). On the contrary, white cream was the product that obtained the highest density ($\bar{\rho }$ of Blank = 1.04 g/cm^3^), due to the loss of air due to physicochemical instability (the lower the air volume, the higher the density). As for cream with tara gum, it has a density very similar to that of cream with PG, ($\bar{\rho }$ of TG = 0.99 g/cm^3^), which indicates that it can also be an alternative as a substitute for AG and PG from the approach to this food quality. 

Creams formulated with natural gums caused a reduction in gas diffusion from smaller bubbles to larger ones and also reduced lamellar thinning and bubble ruptures. In Table 3, the average value of the diameter of the bubbles is lower, firstly for the cream with tara gum (($\bar{BS}$) of TG = 35 µm), and secondly for the cream with pectin (($\bar{BS}$) of PG = 39 µm). This indicates that tara gum, by improving the viscosity of the colloidal system, also made it possible to stabilize the composition between the liquid phases of the lamellae with the gaseous phase of the bubbles, delaying drainage in the liquid film that separates the bubbles [6]; the smaller the bubble size, the more stable the cream, which is interpreted in the preservation of its smooth and homogeneous texture. On the contrary, it is verified that the cream without gums has the largest size of bubbles (($\bar{BS}$) of 57 µm), on some occasions non-spherical bubbles, and the presence of water masses were seen instead of bubbles, as shown in some images, especially at a temperature of 25 °C. The increase in BS favored by the diffusion phenomenon that allows the bubbles to unite as time passes, and there comes a time when the bubbles may experience thinning due to the drainage of the liquid film that separates them, causing their rupture [22]. When this happens, the field observed in the microscope shows areas with water and fewer bubbles; the effect on the cream caused a heterogeneous texture, with the presence of lumpy masses and also watery masses.

The change in volume and syneresis of the creams containing gums were lower in relation to the creams without gums. The gums, being made up of hydrocolloid-type molecules, improved viscosity, reducing not only the loss of air, but also they reduced the loss of the liquid phase trapped in the food matrix and its consequent induced water evaporation. Table 3 shows that the creams containing TG experienced less volume change, and secondly, the cream with PG (($\bar{\Delta V}$) of TG = 0.80 cm^3^ and ($\bar{\Delta V}$) of PG = 0.94 cm^3^, respectively). It was also found that these gums caused a lower percentage of syneresis ($\bar{S}$ of PG = 8.07% and $\bar{S}$ of TG = 9.31%, respectively). Tara gum, like pectin, reduces the separation of liquid, solid and gaseous phases of the topping creams, stabilizing the system and preserving the homogeneity of the ingredients. On the contrary, the creams that did not contain gums experienced greater loss of volume and also greater syneresis (($\bar{\Delta V}$) of blank = 1.57 cm^3^ and $\bar{S}$ of blank = 23.44%). This is due to the diversity of the ingredients that are susceptible to separating during storage. In addition, it was possible to verify that the carrageenan added to the creams, although they reduced these physicochemical phenomena (loss of volume and syneresis), are relatively greater than those obtained with the other gums (($\bar{\Delta V}$) of CG = 1.18 cm^3^ and $\bar{S}$ of CG = 21.75%), so a good stability of butter-based topping creams could not be ensured.

The decrease in volume can be explained due to the phase separation that the cream undergoes during the storage time, since there is liquid that is released from the matrix of the cream by syneresis of contraction [7]. There is also a rupture of bubbles in the sample due to drainage of the liquid film in addition to the formation of smaller crystals from the butter fat while it melts [6], which produces a smaller final volume. By raising the temperature, the cream loses more structure and therefore loses more volume. In the case of cream made with tara gum, there is less volume loss due to the formation of a network of very stable macromolecules that reduces the phase separation.

The increase in syneresis can be explained because creams are thermodynamically unstable systems, which causes the separation of their phases. The amount of released liquid increased during storage and also when the temperature of the system increased [23]. The observed decrease in syneresis in the previous graphs (Figure 6a,b) could be caused by dehydration of the sample, which causes some sugar to crystallize and a certain amount of liquid to bind to the cream, increasing the difficulty of the syneresis of the system.

It has been shown that the density is closely related to bubble size with a direct relationship as the storage time of the cream passes. However, it must be emphasized that at 25 °C—in a certain time that depends on each cream—the disappearance of bubbles occurs, interrupting the aforementioned relationship. On the other hand, the densities of the creams presented an inverse relationship to their volume, in contrast with the direct relationship between the density and the volume lost by the sample, as indicated by the results presented in this research. The volume of the creams decreased as small bubbles merge to form larger ones because the space occupied by two bubbles is larger than that that of a single bubble [7,9,22].

The creams made TG and PG had the smallest bubble size (($\bar{BS}$) of TG = 35.19 µm and PG = 39.43 µm) as well as the lowest volume loss (($\bar{\Delta V}$) of TG = 0.80 cm^3^ and PG = 0.94 cm^3^), thus demonstrating the direct relationship between these two parameters. As for the cream with AG, the bubble size and the loss of volume are relatively small. However, in the determination of the density, an outstanding performance of this gum was observed because it has a lower density than the rest of creams. This could be explained taking into consideration that since density is closely related to mass and volume, the large amount of air incorporated during beating produces a low-density system which is not sufficiently stable. While time passes, the bubbles fade producing a greater decrease in volume compared with that of creams made with PG and TG. Although GA does not adequately stabilize buttercream, like TG and PG, its low density is an indication of better performance since a greater quantity of the product could be obtained by achieving an adequate whipping process [24].

A well-known property of fats is their capability to crystallize in different ways while maintaining their chemical composition (polymorphism). The crystallization of a fat influences the size and stability of a food product, since an alpha crystal is smaller, less dense, and also less stable than a beta crystal. Additionally, while sudden temperature changes easily liquefy an unstable glass (which melts), slow temperature changes form stable crystals with higher melting points, which is a desired feature in the confectionery industry [6]. The syneresis, such as the reduction in the volume of the cream, is also due to this crystallographic change of the butter fat.

In the rheological analysis, as of day 21, the creams stored at 25 °C—with the exception of the one containing TG—displayed a permanent elastic alteration after being subjected to a rheological analysis. This may be due to the fact that certain gums (arabic, pectin and carrageenan) only thicken the system, while others (for example tara gum) gelify the system at concentrations that are within the range used in this work (0.8%) [24]. This makes tara gum the most suitable for the preparation of butter creams, because during the beating for its preparation the shear will not cause permanent elastic deformation in the cream. 

The increase in hysteresis in the case of creams with tara gum and pectin at 4 °C can be explained due to a change in the texture of the food [25] Meanwhile, the increase in hysteresis at 25 °C in creams with arabic gum, carrageenan or pectin, and in creams without gums, can be originated by a loss of texture in the cream due to the formation of lumps, producing a permanent elastic deformation in the cream [7,26]. In the case of cream with tara gum, the very large value of hysteresis could be due to the formation of a gelled system mentioned above. It was also possible to observe a relationship of the area of the curves with the stability of the creams, which is small for the less stable samples, and large for the more stable creams, i.e., the one made with TG (TI = 74.67 and 1559.90 q.u., at 4 °C and 25 °C, respectively).

The various experimental data obtained in the present work and the corresponding statistical analyses indicate that the phase separation in the creams that were made with the four natural gums was reduced compared to that of the cream that did not have gums. This fact indicates that it was possible to increase the stability of butter creams by adding natural gums, with the additional benefit of improving their texture and nutritional value by providing dietary fiber. The butter cream made with TG showed the best viscosity—it is 5.6 times the one of the cream without gums ($\bar{\eta }$ without gums = 15.49 Pa·s, $\bar{\eta }$ with TG = 87.09 Pa·s), has a bubble size 1.6 times smaller compared to that of the cream without gums (($\bar{BS}$) without gums = 57 μm, ($\bar{BS}$) with TG = 35 μm), a volume loss 2 times lower than that of the cream without gums (($\bar{\Delta V}$) without gums = 1.57 cm^3^, (($\bar{\Delta V}$) with TG = 0.80 cm^3^), a rheology with thixotropic behavior without permanent elastic deformation and higher thixotropic index when compared to those of the cream without gums (cream without gums with plastic and thixotropic behavior, with permanent elastic deformation) (TI max. without gums = 17.40 y 71.78 q.u., TI max. with TG = 74.67 and 1559.90 q.u., at 4 °C and 25 °C, respectively).

## 5. Conclusions

The physicochemical properties of the coverage creams for cake formulated with natural gums were evaluated, in which it was found that tara gum has thickening and stabilizing properties, which are superior to those of gum arabic, carrageenan and pectin. The viscosity and thixotropic index evaluated in the creams were higher in those that contained tara gum, in addition, they implied smaller bubble size and lower volume change in the storage conditions of 4 and 25 °C for 28 days. Carrageenan was the gum that had the least influence on the physicochemical properties compared to the rest of gums, so it is not recommended for the preparation of coverage creams for butter-based cakes. The addition of tara gum reduces the phase separation of the coverage creams for cake, which indicates a positive effect in reducing the loss of texture, retention of the ingredients in the food matrix and maintains the quality of the product both in terms of nutritional properties, by having a natural gum rich in dietary fiber, and in terms of sensory quality by preserving a homogeneous visual appearance for consumers.

## Figures and Tables

**Table 1:** Research factors and variables.

Factors	Variables
Cream formulation	Absence of gums
	(Blank)
	Arabic gum (0.8%)
	Tare gum (0.8%)
	Carrageenan (0.8%)
	Pectin (0.8%)
Temperature	25 °C
	4 °C
Time	0 days
	3 days
	7 days
	10 days
	14 days
	17 days
	21 days
	24 days
	28 days

**Table 2:** Sensory evaluation. Preference test.

Method	Number of Trials	Confidence Level 99% (Two-Tailed Test)
M1	11	
M2	69	
Total	80	52

**Table 3:** Estimated marginal means evaluated in the Tukey test.

Estimated Marginal Means *						
Formulation	N	Viscosity (Pa·s)	Density (g·cm^−3^)	Bubble Size (µm)	Lost Volume (cm^3^)	Syneresis (%)
Blank	54	15.49 ± 1.81	1.04 ± 0.01	57 ± 8	1.57 ± 0.27	23.44 ± 4.59
CG	54	16.09 ± 1.35	1.01 ± 0.02	49 ± 8	1.18 ± 0.21	21.75 ± 4.40
AG	54	35.00 ± 6.72	0.96 ± 0.02	47 ± 7	0.98 ± 0.17	17.12 ± 4.14
PG	54	41.63 ± 3.93	0.99 ± 0.02	39 ± 6	0.94 ± 0.16	8.07 ± 2.08
TG	54	87.09 ± 24.19	0.99 ± 0.02	35 ± 6	0.80 ± 0.16	9.31 ± 1.89

* Measurements expressed with the standard error at a confidence level of 95% (*p* < 0.05).

**Table 4:** Rheological behavior of the curves τ vs. D and η vs. D.

Day	Blank		AG		TG		CG		PG	
	4 °C	25 °C	4 °C	25 °C	4 °C	25 °C	4 °C	25 °C	4 °C	25 °C
0	P	P	T	T	P	P	P	P	P	P
7	P	T	T	T	T	T	P	T	T	T
14	P	T	T	T	T	T	P	T	T	T
21	P	T	T	T	T	T	P	T	T	T
28	P	T	T	T	T	T	P	T	T	T

P: plastic; T: thixotropic.

**Table 5:** Summary of measured parameters in which the gums have played a relevant stabilizing function considering the significant difference.

Parameter	Natural Gums			
	AG	TG	CG	PG
Viscosity		x		
Density	x			
Bubble size		x		x
Volume		x		
Syneresis				x
Rheology		x		

## Data Availability

The data are available in the Library of the Faculty of Chemical Sciences of the Universidad Central del Ecuador.
